# Combination of Coagulation and Ozone Catalytic Oxidation for Pretreating Coking Wastewater

**DOI:** 10.3390/ijerph16101705

**Published:** 2019-05-15

**Authors:** Lei Chen, Yanhua Xu, Yongjun Sun

**Affiliations:** 1 School of Environmental Science and Engineering, Nanjing Tech University, Nanjing 211800, China; yanhuaxu18@hotmail.com; 2 College of Urban Construction, Nanjing Tech University, Nanjing 211800, China

**Keywords:** coking wastewater, ozone catalysis, coagulation, combined process

## Abstract

In this study, coagulation, ozone (O_3_) catalytic oxidation, and their combined process were used to pretreat actual coking wastewater. The effects on the removal of chemical oxygen demand (COD) and phenol in coking wastewater were investigated. Results showed that the optimum reaction conditions were an O_3_ mass flow rate of 4.1 mg min^−1^, a reaction temperature of 35 °C, a catalyst dosage ratio of 5:1, and a O_3_ dosage of 500 mg·L^−1^. The phenol removal ratio was 36.8% for the coagulation and sedimentation of coking wastewater under optimal conditions of 25 °C of reaction temperature, 7.5 reaction pH, 150 reaction gradient (G) value, and 500 mg·L^−1^ coagulant dosage. The removal ratios of COD and phenol reached 24.06% and 2.18%, respectively. After the O_3_-catalyzed oxidation treatment, the phenols, polycyclic aromatic hydrocarbons, and heterocyclic compounds were degraded to varying degrees. Coagulation and O_3_ catalytic oxidation contributed to the removal of phenol and COD. The optimum reaction conditions for the combined process were as follows: O_3_ dosage of 500 mg·L^−1^, O_3_ mass flow of 4.1 mg·min^−1^, catalyst dosage ratio of 5:1, and reaction temperature of 35 °C. The removal ratios of phenol and COD reached 47.3% and 30.7%, respectively.

## 1. Introduction

Coking wastewater is generated by coal coking and mainly includes residual ammonia water, gas cold sewage, and sewage generated by chemical product refinement [1]. Coking wastewater is a series of phenolic substances, cyanide, petroleum substances, sulfides, ammonia nitrogen and other organic compounds emitted during the coking industry. The industrial wastewater has different water quality depending on the nature of the raw coal used in the production process, the processing technology (including coking and product recovery process), and the carbonization temperature. Generally, coking wastewater can be divided into high-concentration organic wastewater and high-solids suspended wastewater according to wastewater quality. High-concentration organic wastewater, also known as phenol-containing wastewater, mainly contains phenol, cyanide, benzene, ammonia, oil, and high solid suspension. The remaining ammonia water contains high concentrations of ammonia, phenol, cyanide, and oil, which are the main sources of coking wastewater [2]. The remaining ammonia water is combined with direct cooling water for gas cooling, direct steam condensation separation water for crude benzene processing, direct steam condensation separation water for tar refinement, and washing water containing phenol and cyanide [3]. The wastewater of sulfide and oil is collectively referred to as phenol cyanide wastewater. This wastewater has large amounts of water and a complex composition, and it is a typical refractory wastewater in the coking industry. The main organic substances in coking wastewater are phenols, benzenes, heterocyclic compounds, and polycyclic compounds [4]. Among these substances, phenolic compounds, which include phenol, o-methylphenol, p-methylphenol, and dimethylphenol, have the highest content. Benzene and its derivatives include benzene, toluene, xylene, naphthalene, anthracene, phenanthrene, and benzopyrene [5]. Heterocyclic compounds include quinoline, pyridine, hydrazine, carbazole, furan, and thiophene. The concentration of salt in coking wastewater can reach several thousands of mg·L^−1^, in which inorganic substances mainly include ammonia nitrogen, sulfate, chloride, carbonic acid (hydrogen), thiocyanate, cyanide-containing compounds (cyanide and ferrocyanide), and sulfur ions [6]. High salt content, especially high ammonia nitrogen, has a strong inhibitory effect on microbial bacterial activity and thus increases the difficulty of biological nitrogen removal [7].

Currently, coking wastewater treatment still uses biological procedures. However, direct biological treatment exerts a serious impact on the biochemical pool and eventually causes the death of microorganisms due to the complex composition and high toxicity of coking wastewater [8]. Therefore, pretreatment of coking wastewater is crucial. Many pretreatment techniques, such as coagulation and ozone (O_3_) catalytic oxidation, are available. Pretreatment technology is generally selected in accord with the treated object [9]. Common methods for removing suspended matter and oil substances include oil separation/precipitation, air floatation, and coagulation sedimentation methods [10]. Ozone-based processes do not always lead to a complete mineralization of compounds [11]. The performance of the UV/O3 process leads to the increase of the toxicity of post-processed water solutions [12]. When organic matter in water, such as heterocyclic and biotoxic organic compounds (some of which are dissolved in wastewater), is difficult to degrade, it is commonly pretreated by advanced oxidation, iron–carbon microelectrolysis, ultrasonic oxidation, and Fenton oxidation to overcome difficult degradation effectively [13]. The destroyed molecular structure of organic matter improves the biodegradability of wastewater [14]. This study combines coagulation and O_3_ catalytic oxidation because of the complex composition of coking wastewater, and such a combination is expected to achieve a good treatment effect.

Coagulation is a commonly used method in water treatment [15]. Coagulation utilizes a chemical agent that converts fine contaminants that are stably dispersed in water into a destabilized state and aggregates them into a mixture or floc that is easy to separate for the removal of pollutants [16]. Enhanced coagulation has elicited considerable attention in the field of water treatment. Research on enhanced coagulation has focused on the development of new coagulants and optimization of coagulation conditions [17]. O_3_ catalytic oxidation is an advanced process that can promote the decomposition of O_3_ to produce non-selective hydroxyl radicals [18]. It can effectively address the low removal ratio of refractory organic pollutants and improve wastewater mineralization and O_3_ utilization rates [19]. Thus, this process has attracted considerable attention in the advanced treatment of industrial wastewater. O_3_ catalytic oxidation effectively removes refractory and biotoxic organic substances, such as phenols, polycyclic aromatic hydrocarbons, and nitrogen-containing heterocyclic compounds, in coking boiling water [20]. It facilitates decolorization, deodorization, and sterilization and does not cause secondary pollution. Additionally, O_3_ catalytic oxidation is simple to implement and easy to manage. O_3_ oxidation exerts a good removal effect on refractory chemical oxygen demand (COD) [21]. The purpose of this study is to pretreat the hard-to-biodegradable coking wastewater by ozone catalytic oxidation technology, so that some organic matter can be effectively degraded, the biological toxicity of wastewater can be effectively reduced, and the biodegradable macromolecular organic matter can be degraded and destroyed. The biodegradability of coking wastewater is greatly improved, laying the foundation for the subsequent biochemical process. Ozone oxidation technology can overcome the problem that the traditional Fenton technology needs to adjust the pH to increase the salt content of the wastewater. In addition, we want to make ozone the catalyst to overcome the problem involving the conventional ozone catalyst, which is easily poisoned in the actual wastewater.

Steel industries are often built near large rivers and lakes because of the large amount of water used in their production and the production of large amounts of sewage. The coking wastewater produced by the steel plant is finally treated and discharged to the natural water body by < Discharge standard of pollutants for municipal wastewater treatment plant > (GB 18918-2002). If the discharged water after wastewater treatment cannot meet the requirement of discharge standard, the water quality of the basin will be polluted and deteriorated, causing deterioration of water quality and ecological damage. Pollution control of large industrial enterprises on both sides of the river is the focus of healthy watershed management. The control of pollutants entering into the river can be achieved by controlling the drainage water quality of enterprises on both sides of the river. Therefore, the advanced treatment of coking wastewater is strengthened to meet the discharge standard. This has positive implications for healthy watershed management and point source pollution control.

In this study, coagulation, O_3_ catalytic oxidation, and their combinations were used to pretreat coking wastewater. The effects of O_3_ oxidation and O_3_ catalytic oxidation on coking wastewater were compared. The effects of catalyst dosage ratio, O_3_ dosage, O_3_ mass flow rate, reaction temperature, and hydrogen peroxide (H_2_O_2_) dosage on the catalytic oxidation performance of O_3_ were evaluated. Moreover, the effect of coking wastewater on a separate coagulation process was assessed. The treatment effect of combined coagulation + O_3_ catalytic oxidation on coking wastewater was investigated, and the possible degradation mechanism was analyzed through ultraviolet (UV) spectroscopy and gas chromatography–mass spectrometry (GC–MS).

## 2. Materials and Methods

### 2.1. Experimental Materials

The experimental wastewater used in this study was obtained from the ammonia distillation tower of a coking plant in Zhangjiagang City, Jiangsu Province. COD reached 3900–4200 mg·L^−1^, the chroma was more than 500 times, and pH was 10 ± 0.5. The raw water quality of coking wastewater is shown in Table 1.

The experimental reagents, namely, phenol, concentrated sulfuric acid, silver sulfate, ammonium ferrous sulfate, potassium dichromate, ammonium chloride, ammonia, potassium ferricyanide, sodium thiosulfate, sodium hydroxide, sodium chloride, anhydrous sodium sulfate, and 4-amino reagents (e.g., ntipyrine and ammonium chloride), were of analytical grade. Phenol was chromatographically pure. These experimental reagents were produced by Sinopharm Group Shanghai Chemical Reagent Co., Ltd. O_3_ was prepared on-site by using a Ozone generator (Ozone generator, CF-G-3-010g, Qingdao Guolin Industrial Co., Ltd., Qingdao, China) O_3_ generator, with high-purity oxygen (99.99%) as the gas source.

### 2.2. Test Methods

An O_3_ generator (Ozone generator, CF-G-3-010g, Qingdao Guolin Company, Qingdao, China) and an O_3_ detector (LontecLT-200B, Qingdao Langke Electronic Technology Co., Ltd., Qingdao, China) using pure oxygen were employed in this study. O_2_/O_3_ mixed gas was generated by the O_3_ generator for the gas source. The O_2_/O_3_ mixed gas was placed in a homemade plexiglass reactor in a digital thermostatic water bath (85-2, Jiangsu Jinyi Instrument Technology Co., Ltd., Changzhou, China). An electronic balance (P224, Sartorius, Göttingen, Germany) was used to measure a certain amount of catalyst solids, and a cylinder was adopted to obtain a certain amount of wastewater. A peristaltic pump (YZ1515X, Lange Constant Flow Pump Co., Ltd., Baoding, China) was utilized to recycle the wastewater inside the O_3_ reactor. The same electronic balance was utilized to weigh a certain amount of coagulant, which was added with a specific amount of distilled water and made to pass through a temperature-controlled magnetic stirrer (HH-S1, Jiangsu Gold Yi Instrument Technology Co., Ltd., Huaian, China). Stirring was performed for 10 min to obtain a coagulant. The pretreatment of coking wastewater through O_3_ catalytic oxidation was investigated via a single-factor optimization test to determine the effect of catalyst addition amount, O_3_ dosage, H_2_O_2_ dosage, and initial reaction temperature on the treatment of phenol in distilled ammonia wastewater. In optimal reaction conditions, coagulation and sedimentation pretreatment of coking wastewater were investigated via a single-factor optimization test to assess the effect of single and composite coagulants on COD and phenol treatment in steamed ammonia wastewater and determine the best response in the single-factor optimization test. Then, the O_3_ catalytic oxidation test, coagulation reaction experiment, and H_2_O_2_ under optimal conditions were combined to determine the optimal reaction conditions of the combined process. The experimental values are the average of three experiments with a relative error of less than 10%.

Water quality analysis was conducted using standard methods. The concentration of the phenol solution was determined with a UV spectrophotometer. The absorbance of the standard-concentration phenol solution was determined with a UV-visible spectrophotometer (UV2600, Shimadzu Corporation, Kyoto, Japan). The phenol concentration for the concentration–absorbance standard curve was determined based on the absorbance and standard curve of the phenol solution to be tested. COD was determined based on the national standard GB 11914-1989, and the color of wastewater was determined based on the national standard GB11903-89.

## 3. Results and Discussions

### 3.1. O_3_ Catalytic Oxidation Treatment of Coking Wastewater

#### 3.1.1. Effect of Catalyst and O_3_ on O_3_ Catalytic Oxidation and Phenol Removal

**Figure 1:**
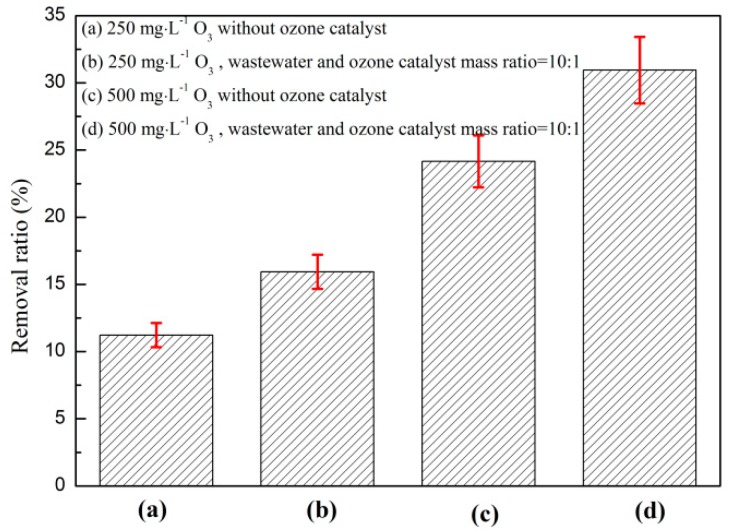
Effect of catalyst and O_3_ on coking wastewater treatment via ozone catalytic oxidation.

The effect of the catalyst and O_3_ on the catalytic oxidation of O_3_ to phenol is shown in Figure 1. The test conditions were as follows: The starting phenol concentration of the wastewater was 1060 mg·L^−1^, the reaction temperature was 25 °C, and the O_3_ mass flow rate was 2 mg·min^−1^. As shown in Figure 1, the phenol removal ratio could reach up to 11.2% with the increase in reaction time when no catalyst was added to the reaction system and the O_3_ dosage was 250 mg·L^−1^. When the O_3_ dosage increased to 500 mg·L^−1^, the removal efficiency of phenol increased significantly, and the highest removal ratio was 24.2%. The removal ratio of phenol was remarkably improved when a catalyst was added to the reaction system. The removal ratio of O_3_ was 15.9% when the dosage was 250 mg·L^−1^, and the removal ratio was 30.9% when the O_3_ dosage was 500 mg·L^−1^.

O_3_ is the direct driving force of the degradation of organic matter, and its dosage directly affects the rate and effect of its catalytic oxidation [22]. As shown in Figure 1, the removal effect of phenol on the reaction system was remarkably improved when the O_3_ dosage was increased. O_3_ can directly react with organic substances in water in the form of molecules, and an increase in O_3_ dosage can accelerate the reaction [23]. The removal ratio of phenol rapidly increased when the catalyst was added to the reaction system because the catalyst offered an active site for the catalytic oxidation of O_3_, which could provide a three-phase reaction interface by adsorbing O_3_, water, and organic matter and increase O_3_ doping. The amount was beneficial to the mass transfer of the three-phase interface, which accelerated the reaction rate and further increased the removal ratio of phenol in the coking wastewater of the reaction system [24]. In summary, the catalyst is an indispensable factor of the O_3_ catalytic oxidation reaction system. Increasing the O_3_ dosage aids in improving the removal ratio of phenol.

#### 3.1.2. Effect of Catalyst Dosage Ratio on O_3_ Catalytic Oxidation Performance of Phenol Removal

**Figure 2:**
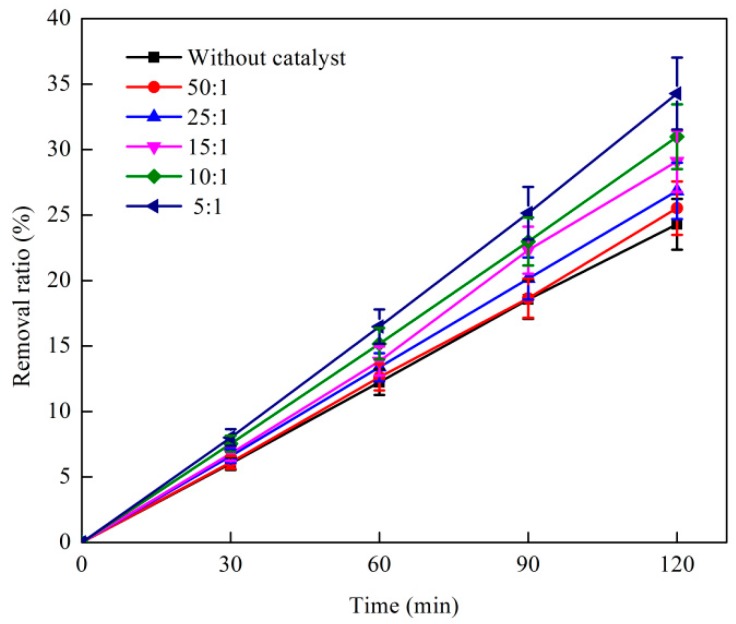
Effect of catalyst dosage ratio on coking wastewater treatment by O_3_ catalytic oxidation.

The effect of catalyst dosage ratio on the catalytic removal of phenol by O_3_ oxidation is shown in Figure 2. The test conditions were as follows: The initial phenol concentration of the wastewater was 1060 mg·L^−1^, the O_3_ dosage was 500 mg·L^−1^, the O_3_ mass flow rate was 4.1 mg·min^−1^, and the reaction temperature was 25 °C. As shown in Figure 2, the removal efficiency of phenol in the reaction system increased with the increase in catalyst addition ratio. The removal ratio of phenol was optimal when the catalyst dosage ratio was increased to 5:1, which is 34.3%.

As shown in Figure 2, the removal ratio of phenol rapidly increased with the increase in the volume ratio of the catalyst probably because the initial concentration of phenol was high, and the reaction system was in the case where the O_3_ dosage was unchanged [25]. The effective site of the surface increased with the increase in catalyst dosage and helped in catalyzing O_3_ in order to produce a high concentration of OH, which made the reaction thorough. In summary, the optimum catalyst dosage ratio of the O_3_ catalytic oxidation reaction system was 5:1.

#### 3.1.3. Effect of O_3_ Mass Flow on O_3_ Catalytic Oxidation Performance of Phenol Removal

**Figure 3:**
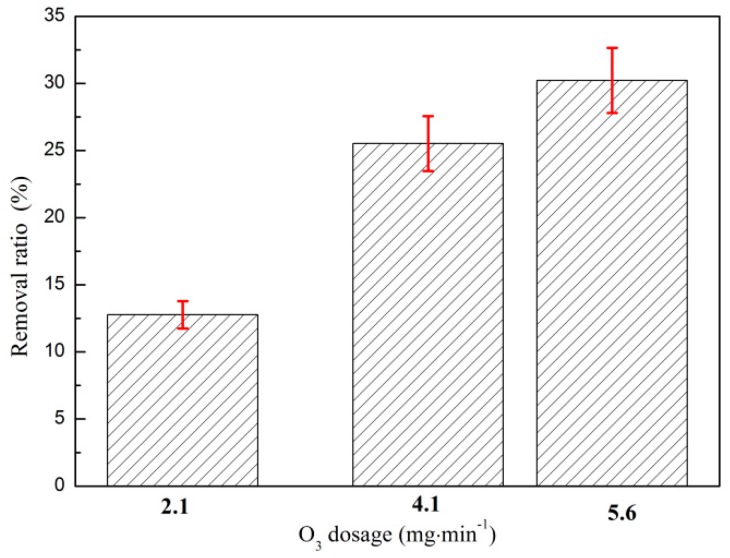
Effect of different O_3_ dosage rate on coking wastewater treatment by O_3_ catalytic oxidation.

The effect of O_3_ dosage rate on the catalytic removal of phenol by O_3_ is shown in Figure 3. The test conditions were as follows: The initial phenol concentration of the wastewater was 1060 mg·L^−1^, the O_3_ dosage was 500 mg·L^−1^, the catalyst dosage ratio was 5:1, and the reaction temperature was 25 °C. As shown in Figure 3, the removal ratio of phenol increased with the decrease in O_3_ dosage rate, but the reaction equilibrium time was extended. Phenol removal occurred when the O_3_ mass flow rate was 5.6 mg·min^−1^ and the reaction time was 90 min. The rate of phenol removal was 35.2% when the mass flow rate of O_3_ was 2.1 mg·min^−1^ and the reaction time was 240 min. The phenol removal ratio was 34.8% when the mass flow rate of O_3_ was 4.1 mg·min^−1^ and the reaction time was 120 min.

The removal ratio of phenol increased with the decrease in O_3_ dosage rate. However, the reaction rate significantly decreased, and the removal ratio was stable. When the O_3_ dosage rate was too large, it accelerated the reaction rate and made the reaction reach equilibrium quickly; however, it easily caused the O_3_ that participated in the reaction to directly overflow from the reaction system. Conversely, the decrease in O_3_ dosing rate was beneficial to the increase in O_3_ [26]. The reaction time between water and organic matter, that is, in three phases, made the reaction thorough [27]. Considering the economic cost and time benefit, the optimal O_3_ mass flow rate of the O_3_ catalytic oxidation reaction system was 4.1 mg·min^−1^.

#### 3.1.4. Effect of O_3_ Dosage on O_3_ Catalytic Oxidation Performance of Phenol Removal

**Figure 4:**
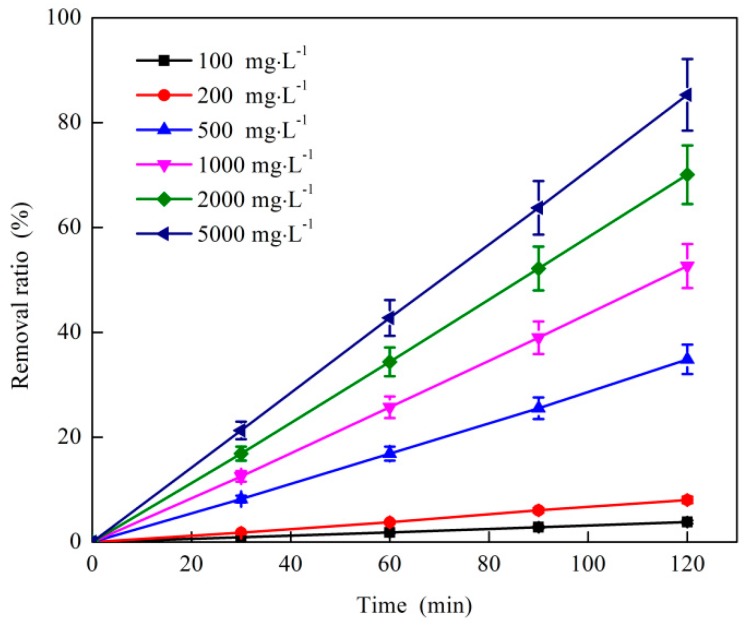
Effect of O_3_ dosage on coking wastewater treatment by O_3_ catalytic oxidation.

The effect of O_3_ dosage on the catalytic removal of phenol by O_3_ is shown in Figure 4. The test conditions were as follows: the starting phenol concentration of the wastewater was 1060 mg·L^−1^, the O_3_ mass flow rate was 4.1 mg·min^−1^, the catalyst dosage ratio was 5:1, and the reaction temperature was 25 °C. As shown in Figure 4, the increase in phenol removal was not obvious with the increase in O_3_ dosage. The phenol removal ratio steadily rose with the increase in O_3_ dosage. The phenol removal ratio reached 34.8% when the dosage was 500 mg·L^−1^. The increase in phenol removal ratio was reduced by the increase in O_3_ dosage.

As shown in Figure 4, the removal ratio of phenol increased with the increase in O_3_ dosage because the content of •OH in the reaction system was remarkably increased by the increase in O_3_ concentration, which was beneficial to the sufficient contact of •OH with organic molecules [28]. It promoted the cleavage of macromolecular chains and groups, such as benzene rings. Thus, macromolecular substances were easily oxidized into small molecular substances, thereby improving the removal effect of phenol [29]. The growth rate of the phenol removal ratio was reduced by the increase in O_3_ dosage because the content of phenol in the reaction system decreased as the reaction progressed, and the collision probability of •OH formed by O_3_ decreased [30]. The possibility of reaction with other substances increased, resulting in a decrease in rate. Taking into account the economic cost, the optimal O_3_ dosage of the O_3_ catalytic oxidation reaction system was 500 mg·L^−1^.

#### 3.1.5. Effect of Reaction Temperature on O_3_ Catalytic Oxidation Performance of Phenol Removal

**Figure 5:**
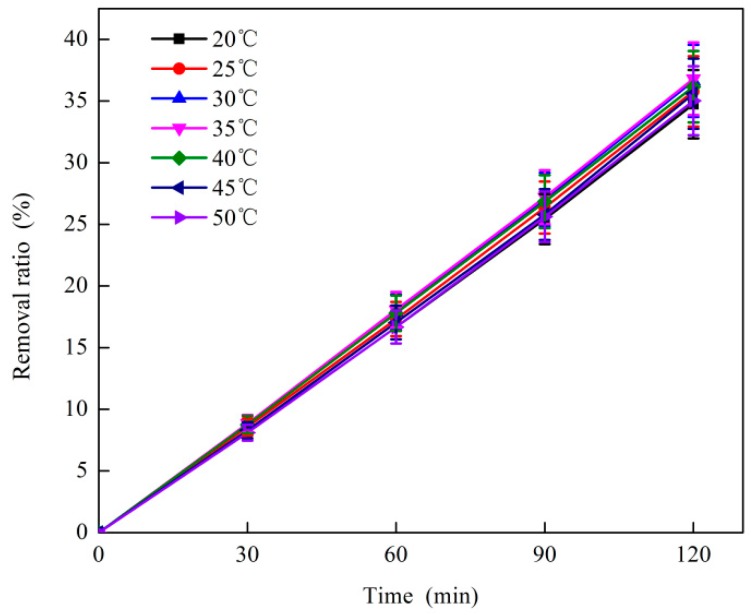
Effect of reaction temperature on O_3_ catalytic oxidation of coking wastewater.

The effect of reaction temperature on O_3_ catalytic oxidation to phenol is shown in Figure 5. The test conditions were as follows: the initial phenol concentration of the wastewater was 1060 mg·L^−1^, the O_3_ dosage was 500 mg·L^−1^, the O_3_ mass flow rate was 4.1 mg·min^−1^, and the catalyst dosage ratio was 5:1. As shown in Figure 5, the removal effect of phenol initially increased then decreased with the increase in reaction temperature. The optimum removal ratio of phenol was 36.8% when the reaction temperature was 35 °C.

As shown in Figure 5, the removal ratio of phenol increased then decreased with the increase in reaction temperature. An appropriate reaction temperature can increase the activity of hydroxyl radicals, increase the degradation rate of phenol in the reaction system, increase the reaction temperature to some extent, help reduce the activation energy of the catalytic degradation reaction, and make the reaction of OH and organic matter complete [31]. Continuous increase in reaction temperature aggravates the decomposition effect of O_3_, resulting in a decrease in the actual O_3_ concentration and a decrease in the removal ratio of phenol [32]. In summary, the optimum reaction temperature for the O_3_ catalytic oxidation reaction system was 35 °C.

#### 3.1.6. Effect of H_2_O_2_ Dosage on O_3_ Catalytic Oxidation Performance of Phenol Removal

**Figure 6:**
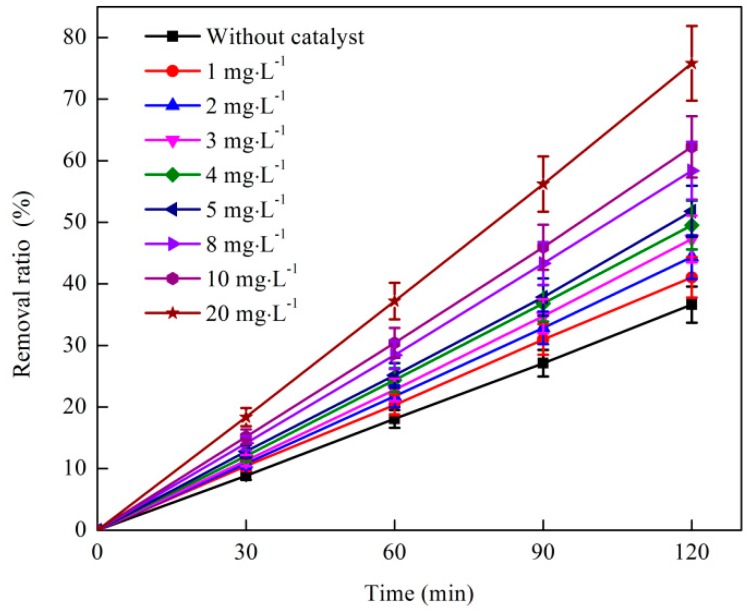
Effect of H_2_O_2_ dosage on coking wastewater treatment by O_3_ catalytic oxidation.

The effect of H_2_O_2_ dosage on the catalytic removal of phenol by O_3_ is shown in Figure 6. The test conditions were as follows: the initial phenol concentration of the wastewater was 1060 mg·L^−1^, the O_3_ dosage was 500 mg·L^−1^, the O_3_ mass flow rate was 4.1 mg·min^−1^, the catalyst dosage ratio was 5:1, and the reaction temperature was 35 °C. As shown in the figure, the addition of H_2_O_2_ to the O_3_ catalytic oxidation reaction system effectively promoted the removal of phenol. The removal ratio of phenol was remarkably improved with the increase in H_2_O_2_ dosage, but the growth rate did not show a large increase. The phenol removal ratio increased from 41.1% to 58.4%, the H_2_O_2_ dosage increased to 10.0 mg·L^−1^, and the phenol removal ratio increased by 3.8% with the increase in H_2_O_2_ dosage from 1.0 mg·L^−1^ to 8.0 mg·L^−1^.

As shown in Figure 6, the increase in H_2_O_2_ dosage within a certain range accelerated the degradation rate of phenol because H_2_O_2_ reacts with Fe^3+^ in the reaction system to form Fe^2+^. Fe^2+^ can further form •OH with H_2_O_2_, and H_2_O_2_ and O_3_ can form •OH, which remarkably increases the concentration of •OH in the solution and promotes the degradation of phenol [33]. Excess H_2_O_2_ that participates in the reaction has a certain degree of reducibility when the H_2_O_2_ dosage exceeds a certain amount [34]. Excess H_2_O_2_ inhibits the removal of phenol from the reaction system. In summary, the optimal H_2_O_2_ dosage of the O_3_ catalytic oxidation reaction system was 8.0 mg·L^−1^.

### 3.2. Coagulation for Coking Wastewater Treatment

#### 3.2.1. Effect of a Single Coagulant on the Removal of COD and Phenol by Coagulation

**Figure 7:**
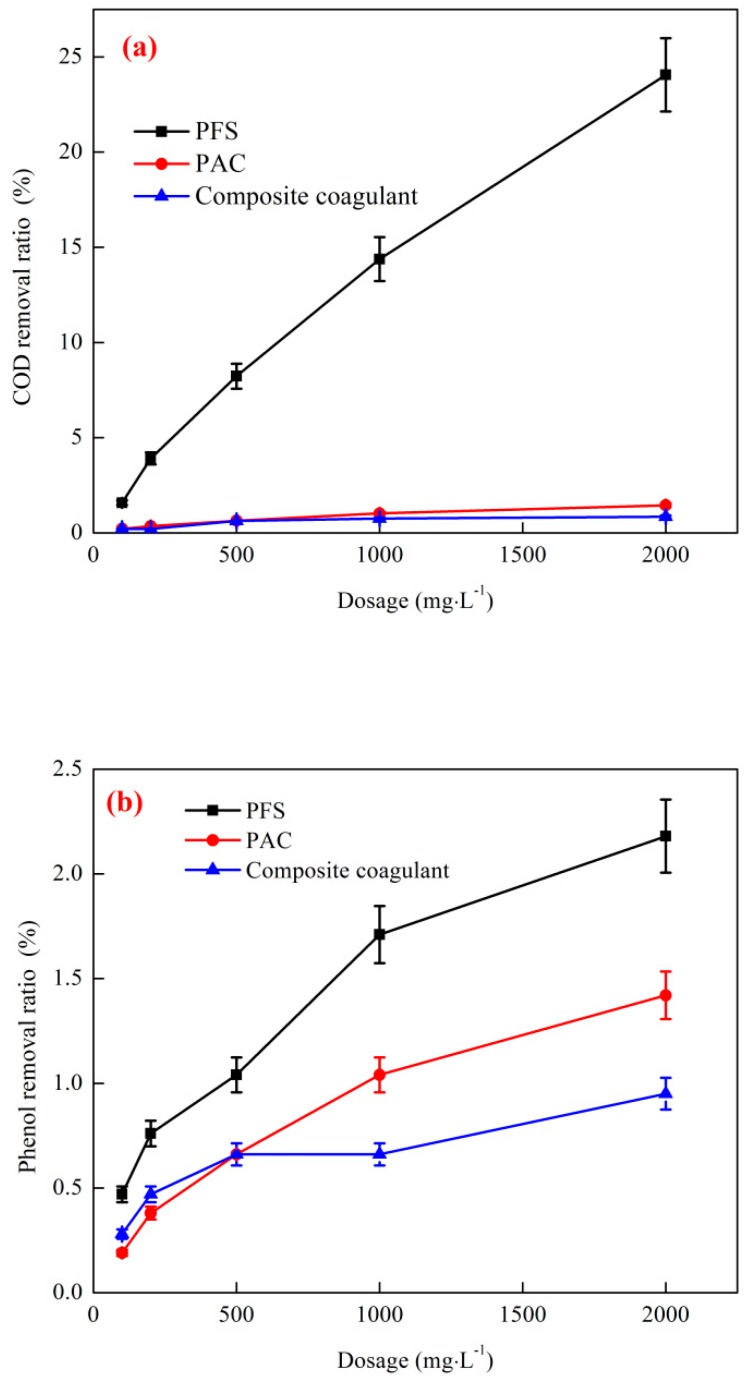
Effect of different coagulants on the coagulation of coking wastewater. (**a**) COD removal; (**b**) Phenol removal.

The effect of a single coagulant on the removal of COD and phenol by coagulation is shown in Figure 7. The test conditions were as follows: the starting phenol concentration of the wastewater was 1053 mg·L^−1^, the COD concentration was 4812 mg·L^−1^, the reaction temperature was 25 °C, the reaction pH was 7.5, and the reaction G value was 150. The removal effect of phenol was poor, but a high COD removal ratio was achieved when coking wastewater was treated with a single coagulant. In the comparative test, when compared with the use of polyaluminum chloride (PAC) and composite coagulant coagulants, the use of polyferric sulfate (PFS) as a coagulant achieved better treatment results, and the removal ratios of COD and phenol were 24.06% and 2.18%, respectively.

As shown in the figure, PFS exhibited the best coagulation effect and a good removal effect on COD and phenol probably because Fe^3+^ has the effect of flocculation and sedimentation and possesses certain oxidative properties that can oxidize and degrade organic matter in coking wastewater, thereby reducing its COD content [35]. PAC and composite coagulants do not have the corresponding oxidation properties. In summary, PFS is the best option when using a single coagulant to treat coking wastewater [36].

#### 3.2.2. Effect of a Composite Coagulant on the Removal of COD and Phenol by Coagulation

**Figure 8:**
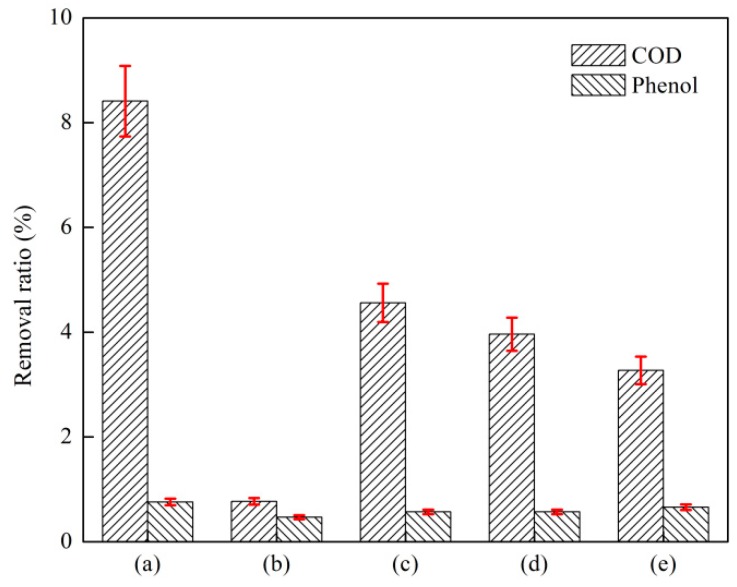
Effect of composite coagulant on coagulation and sedimentation treatment of coking wastewater (**a**) polyferric sulfate (PFS); (**b**) polyaluminum chloride (PAC); (**c**) PFS: Polyacrylamide (PAM) = 5:1; (**d**) PFS:PAM = 10:1; (**e**) PFS:PAM = 15:1).

The effect of a composite coagulant on the removal of COD and phenol by coagulation sedimentation is shown in Figure 8. The test conditions were as follows: The initial phenol concentration of the wastewater was 1053 mg·L^−1^, the COD concentration was 4812 mg·L^−1^, the reaction temperature was 25 °C, the reaction pH was 7.5, the reaction G value was 150, and the coagulant dosage was 500 mg·L^−1^. As shown in the figure, the removal ratio of phenol and COD was low when only PAC was added. The removal of phenol and COD improved when only PFS was added. The removal efficiency of phenol and COD was better than that of PAC when a composite coagulant composed of PFS and polyacrylamide (PAM) was used, but it was not as good as that of PFS. The removal ratio of COD showed a downward trend with the decrease in PAM dosage.

The preparation of a composite coagulant directly affects the treatment effect of coking wastewater. Considering that PAM can remarkably increase the viscosity of a composite coagulant, the PFS solution should be prepared first to avoid PAM wrapping the PFS solid [37]. At the same coagulant dosage, the treatment effect using PFS is obviously due to other coagulants and has a certain decolorization effect because Fe^3+^ has certain adsorption and oxidation capabilities [38]. Considering that PFS is insoluble in PAM and sufficient Fe^3+^ is not released, PAM as a coagulant has no substantial coagulation effect, resulting in a decrease in the treatment effect; however, a certain amount of PAM contributes to the reinforcement [39]. The sedimentation of the body accelerates separation and precipitation [40]. In summary, the ratio of PFS to PAM in the composite coagulant is controlled to some extent for facilitating coagulation and sedimentation treatment of coking wastewater.

### 3.3. Combined Process for the Treatment of Coking Wastewater

#### 3.3.1. Coagulation + O_3_ Catalytic Oxidation Treatment of Coking Wastewater

**Figure 9:**
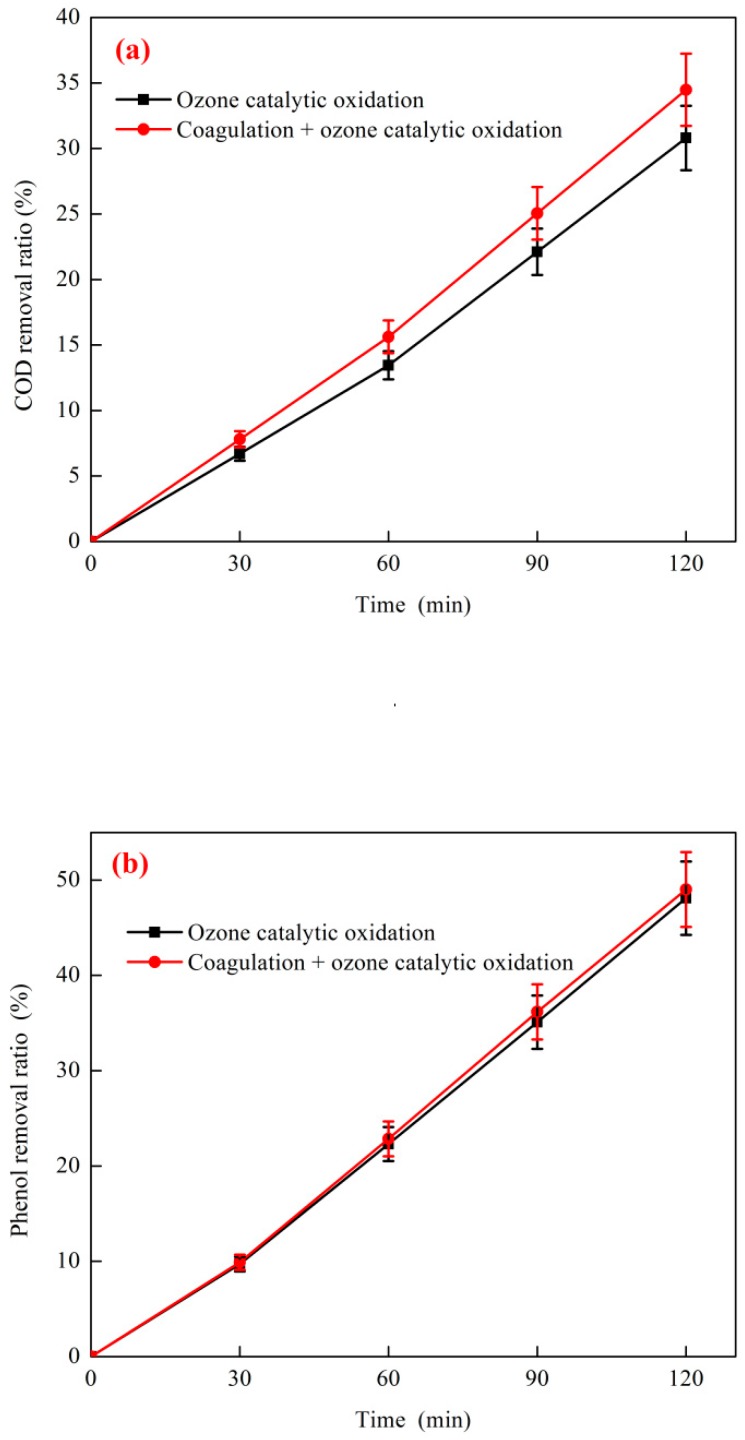
Effect of coagulation on the treatment of coking wastewater by combination process of coagulation + ozone catalytic oxidation: (**a**) COD removal and (**b**) phenol removal.

The effect of coagulation + O_3_ catalytic oxidation on coking wastewater is shown in Figure 9. The coagulation test conditions were as follows: The initial phenol concentration of the wastewater was 1031 mg·L^−1^, the COD concentration was 4881 mg·L^−1^, the reaction temperature was 25 °C, the reaction pH was 7.5, the reaction G value was 150, the dosage of the composite coagulant was 500 mg·L^−1^, and the composite coagulant ratio of Fe_2_SO_4_:PAM was 20:1. The O_3_ catalytic oxidation test conditions were as follows: the O_3_ dosage was 500 mg·L^−1^, the O_3_ mass flow was 4.1 mg·min^−1^, the catalyst dosage ratio was 5:1, the reaction temperature was 35 °C, and the H_2_O_2_ dosage was 3.0 mg·L^−1^. As shown in the figure, the removal ratio of phenol was 48.1% and the removal ratio of COD was 30.8% when coking wastewater was treated by O_3_ catalytic oxidation. The removal ratios of phenol and COD reached 49.0% and 34.5%, respectively, when coking wastewater was treated by combined coagulation + ozone catalytic oxidation. This study used a composite coagulant composed of an inorganic coagulant and an organic polymer coagulant. The price of inorganic–organic composite coagulants is similar to that of traditional inorganic polymer coagulants. However, the flocculation effect is improved, the amount of coagulant is reduced, sludge production is reduced, and the amount of coagulant and the cost of water treatment are reduced due to the enhanced adsorption bridging capacity of inorganic–organic composite coagulants [41]. Compared with organic polymer coagulants, the electric neutralization capability is enhanced, the coagulation effect is improved, and the amount of toxic substances remaining in the water is relatively small due to the small dosage [42]. As shown in the figure, the removal ratio of COD increased significantly after coagulation and sedimentation treatment, which may be because the floc formed by coagulation precipitation can adsorb some of the negatively charged organic macromolecules, which is beneficial to its oxidation into small molecular organics [43]. In summary, the use of coagulation and sedimentation pretreatment before O_3_ catalytic oxidation can improve the removal ratio of phenol and COD in coking wastewater.

#### 3.3.2. Effect of H_2_O_2_ on the Combined Process of Coagulation + O_3_ Catalytic Oxidation

**Figure 10:**
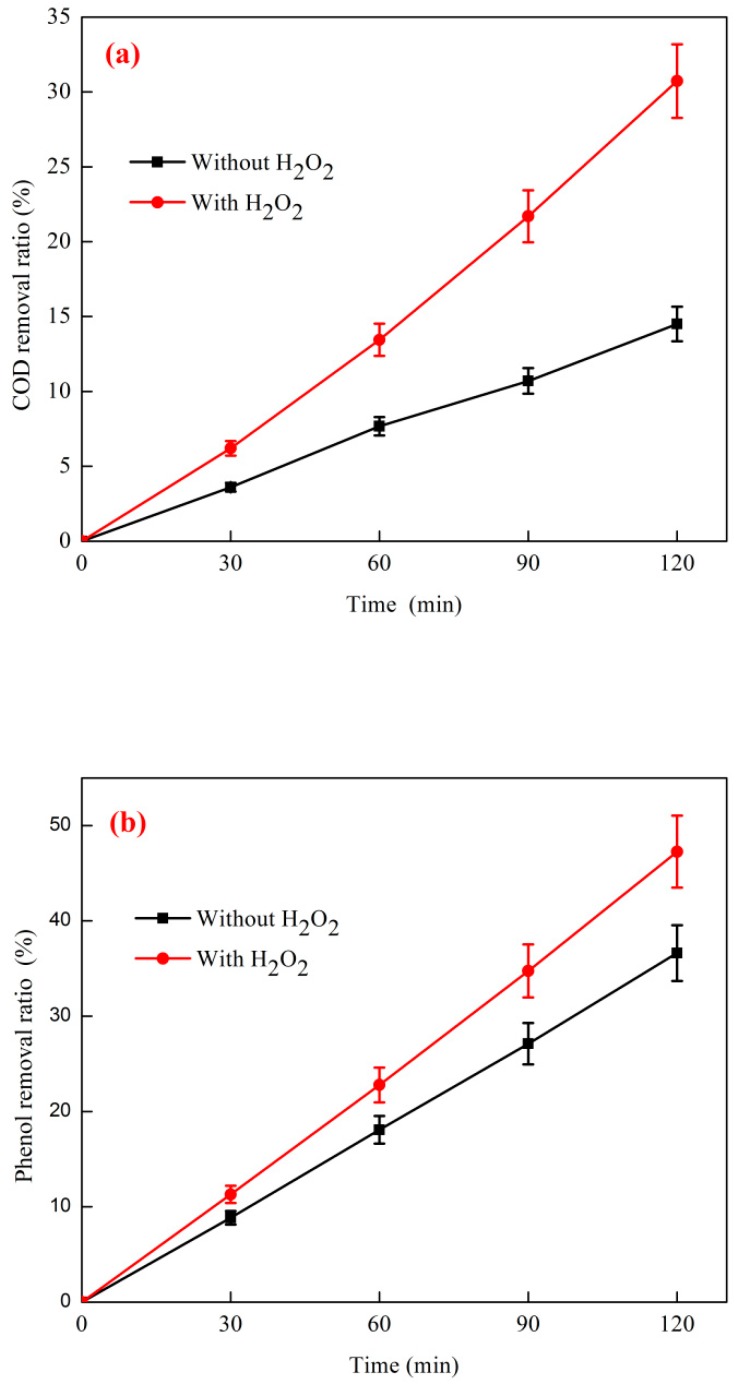
Effect of H2O2 on combined process of coagulation + ozone catalytic oxidation: (**a**) COD removal and (**b**) phenol removal.

The effect of H_2_O_2_ on the combined process of coagulation + O_3_ catalytic oxidation is shown in Figure 10. The experimental conditions of O_3_ catalytic oxidation were as follows: The initial phenol concentration of wastewater was 1044 mg·L^−1^, the COD concentration was 4824 mg·L^−1^, the O_3_ dosage was 500 mg·L^−1^, the O_3_ mass flow rate was 4.1 mg·min^−1^, and the catalyst dosage ratio was 5:1. The reaction temperature was 35 °C, and the H_2_O_2_ dosage was 3.0 mg·L^−1^. The coagulation and sedimentation test conditions were as follows: The reaction temperature was 25 °C, the reaction pH was 7.5, reaction G value was 150, the composite coagulant dosage was 500 mg·L^−1^, and the composite coagulant ratio was Fe_2_SO_4_:PAM = 20:1. As shown in the figure, the removal ratios of phenol and COD in the reaction system were 36.6% and 14.5%, respectively, when H_2_O_2_ was not added. The removal ratios of phenol and COD in the reaction system became 47.3% and 30.7%, respectively, when H_2_O_2_ was added.

As shown in Figure 10, the combined process of coagulation + O_3_ catalytic oxidation was clearly superior to the combined process of coagulation + O_3_ catalytic oxidation, probably because O_3_ catalytic oxidation can effectively remove coking after coagulation precipitation [44]. The particulate suspension and some macromolecular organic matter in the wastewater help reduce the consumption of O_3_ in the particulate suspension and macromolecular organic matter, and the reaction of oxidative degradation of organic matter is thorough [45]. In summary, the addition of H_2_O_2_ helps improve the removal ratio of phenol and COD in coking wastewater, and the best combined process is coagulation + O_3_ catalytic oxidation with the addition of H_2_O_2_.

### 3.4. Mechanism Analysis

#### 3.4.1. UV Analysis

**Figure 11:**
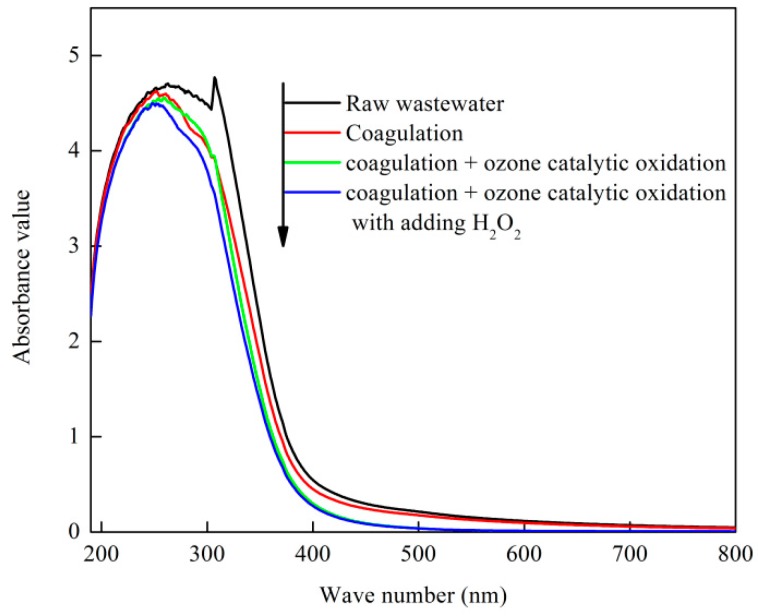
UV-visible spectra of effluent from different treatment processes.

Figure 11 shows the UV-vis spectrum of the coking wastewater raw water and the effluent of each process. As shown in the figure, the UV light absorption of the raw water of coking wastewater was mainly concentrated in the UV region of 190–400 nm. Combined with the source of coking wastewater, we can infer that the main component of the wastewater was aromatic compound. UV254 is a parameter that indicates the content of aromatic compounds in water (including natural organic compounds containing aromatic structures, such as benzenes, phenols, and humus) [46]. UV254 drops the fastest because O_3_ can react with C=C on the aromatic ring, resulting in the ring opening of the aromatic ring. The biodegradability of wastewater is remarkably improved with the oxidative degradation of organic compounds containing benzene rings and macromolecular chain structures [47].

#### 3.4.2. GC–MS Analysis

Figure S1 shows the gas chromatogram of the coking wastewater raw water and the effluent of each process. As shown in the figure, phenols, heterocyclic compounds, polycyclic aromatic hydrocarbons, and their derivatives were the main components of pollutants in the coking wastewater [48]. They can be detected after O_3_ catalytic oxidation. The abundance of most organic compounds decreased to varying degrees, indicating that phenols, polycyclic aromatic hydrocarbons, and heterocyclic compounds were degraded to varying degrees after O_3_-catalyzed oxidation [49]. As shown in Table 2, the composition of raw water is complicated. After the raw water has been coagulated, the composition has remained basically unchanged. The organic substances such as phenol are reduced by Coagulation + O_3_ catalytic oxidation. After Coagulation + O_3_ catalytic oxidation with addition of H_2_O_2_ process, the content of organic substances, such as phenol, is further reduced.

### 3.5. Application of Coagulation and Ozone Catalytic Oxidation in Healthy Watershed Management

In the healthy watershed management, it is extremely important to strengthen point source pollution in the basin. Point source pollution is mainly a relatively concentrated sewage discharge point such as sewage discharge. Its characteristics are concentrated, polluting, destructive and relatively easy to control. If the point source pollutant load exceeds the water environment capacity, it will cause pollution to the water quality of the basin and damage to the watershed ecology. Therefore, internship point source pollution control and end treatment must meet the needs of watershed environmental protection. Additionally, in view of the lack of clean water in rivers, the water quality standards and requirements of sewage treatment plants should be reasonably improved, and the effluent quality of sewage treatment plants should be upgraded to achieve recreational water for reclaimed water. It can be used as one of the ecological water sources of watersheds or lakes, and overall planning of water transfer measures in the outer basin, to achieve the improvement of water environment in the basin and lake under the most acceptable conditions of economic costs. The technology and results of this research are mainly aimed at point source pollution control technology in watershed management and watershed management. Therefore, applying the results of this study to point source pollution control in healthy watersheds has a positive impact.

## 4. Conclusions

In this study, coagulation + O_3_ catalytic oxidation was used to pretreat coking wastewater. In the treatment of coking wastewater by O_3_ catalytic oxidation alone, increasing the O_3_ dosage helped improve the removal ratio of phenol. In the best working condition, the mass flow rate of O_3_ was 4.1 mg·min^−1^, the reaction temperature was 35 °C, and the catalyst dosage ratio was 5:1. The phenol removal ratio was 36.8% when the O_3_ dosage was 500 mg·L^−1^. PFS was more suitable for coking wastewater than PAC and composite coagulant when coking wastewater was treated by coagulation, and the ratio of PFS to PAM in the composite coagulant was controlled to some extent. In the combined process, the use of coagulation and precipitation pretreatment before O_3_ catalytic oxidation helped to improve the removal ratio of phenol and COD in coking wastewater. The addition of H_2_O_2_ significantly improved the treatment effect. The removal ratios of phenol and COD in the O_3_ catalytic oxidation + H_2_O_2_ + coagulation reaction system reached 47.3% and 30.7%, respectively. At this time, the O_3_ dosage was 500 mg·L^−1^. The O_3_ mass flow rate was 4.1 mg·min^−1^, the catalyst dosage ratio was 5:1, the reaction temperature was 35 °C, and the H_2_O_2_ dosage was 3.0 mg·L^−1^. The coagulation sedimentation test conditions were as follows: The reaction temperature was 25 °C, the reaction pH was 7.5, the reaction G value was 150, the composite coagulant dosage was 500 mg·L^−1^, and the composite coagulant ratio was Fe_2_SO_4_:PAM = 20:1. After O_3_-catalyzed oxidation treatment, phenols, polycyclic aromatic hydrocarbons, and heterocyclic compounds were degraded to varying degrees. Hence, O_3_ catalytic oxidation can remarkably improve the biodegradability of wastewater. This study uses the actual coking wastewater as the research object, and the research results can provide theoretical basis and engineering reference for the actual project.

## Figures and Tables

**Table 1:** Raw water quality of coking wastewater.

Index	Characteristic	pH	COD (mg·L^−1^)	Total Nitrogen (mg·L^−1^)	Kjeldahl Nitrogen (mg·L^−1^)	NH_3_-N (mg·L^−1^)	Cyanide (mg·L^−1^)	Salinity (mg·L^−1^)	Total Phenols (mg·L^−1^)	Petroleum (mg·L^−1^)
Value	Yellowish brown, translucent, with a strong pungent odor	9.6	4176	285	118	116	49.3	8500	716	184

**Table 2:** Degradation products list of coking wastewater by Ozone catalytic oxidation pretreatment.

Process	Degradation Products List
Coking wastewater	Phenol, aniline, 2-methylphenol, 3-methylphenol, 2,4-dimethylphenol, 3,5-dimethylphenol, 3,4-dimethylphenol, 2,3-dihydrobenzene And furan, quinoline, anthracene, 1 (2H)-isoquinoline,
Coagulation	Phenol, aniline, 2-methylphenol, 3-methylphenol, 2,4-dimethylphenol, 3,5-dimethylphenol, 3,4-dimethylphenol, 2,3-dihydrobenzene Furan, quinoline, anthracene, 1(2H)-isoquinoline
Coagulation + O_3_ catalytic oxidation	Phenol, 5-methylfurfural, 2-methylphenylhydrazine, 4,5-dimethyl-2-hydroxypyrimidine, rosin acetate, 2,3,4,5-tetramethyl-2-cyclopentenone, 2-methylphenol, 4,5,6-trimethyl-2-pyrimidinone, benzofuran, quinoline, 4-bromo-3-methyl-phenol, 2,3-dihydroindole-4- Alcohol-2-ketone, N-phenylformamide
Coagulation + O_3_ catalytic oxidation with addition of H_2_O_2_	Phenol, 5-methylfurfural, 4,5-dimethyl-2-hydroxypyrimidine, 2,3,4,5-tetramethyl-2-cyclopentenone, 4,5,6 trimethyl-2 -pyrimidinone, 2-hydroxy-4,4-dimethyl-3-indolyl-2-cyclopentanone, N-phenylformamide, quinoline, 2,3-dihydroindole-4-Alcohol-2-ketone

## Supplementary Materials

The following are available online at https://www.mdpi.com/1660-4601/16/10/1705/s1, Figure S1: GC–MS diagram of effluent from different processes: (a) raw water; (b) coagulation; (c) coagulation + O3 catalytic oxidation; (d) coagulation + O3 catalytic oxidation with addition of H2O2.
