# Supplementary material for: Combination of Coagulation and Ozone Catalytic Oxidation for Pretreating Coking Wastewater
Source: Int J Environ Res Public Health. 2019 May 15;16(10):1705. doi: 10.3390/ijerph16101705 (PMC6572503; doi:10.3390/ijerph16101705)
Supplement: Supplementary file 1 [file ijerph-16-01705-s001.pdf]

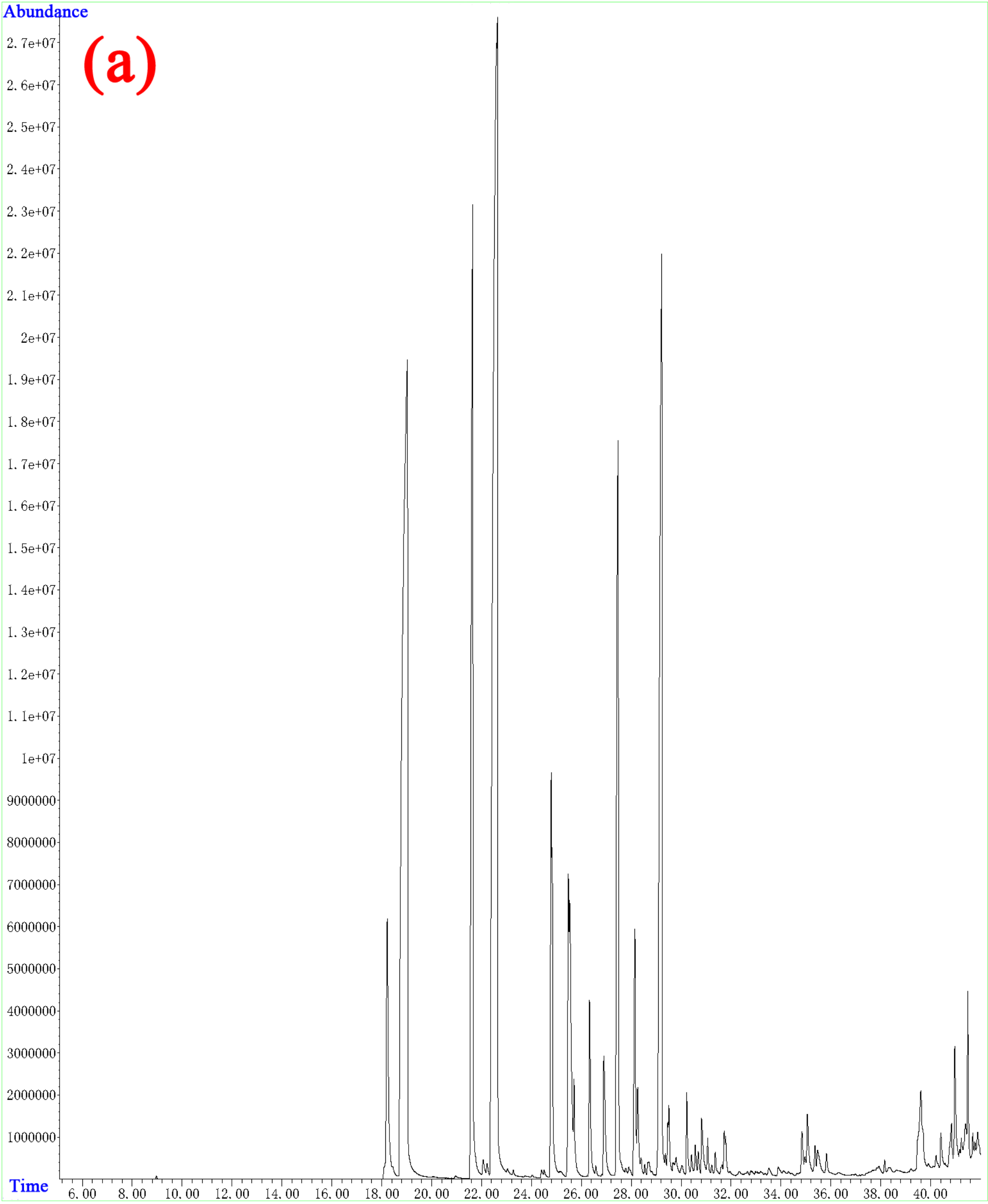

Abundance
(a)
2.7e+07
2.6e+07
2.5e+07
2.4e+07
2.3e+07
2.2e+07
2.1e+07
2e+07
1.9e+07
1.8e+07
1.7e+07
1.6e+07
1.5e+07
1.4e+07
1.3e+07
1.2e+07
1.1e+07
1e+07
9000000
8000000
7000000
6000000
5000000
4000000
3000000
2000000
1000000
Time
6.00
8.00
10.00
12.00
14.00
16.00
18.00
20.00
22.00
24.00
26.00
28.00
30.00
32.00
34.00
36.00
38.00
40.00

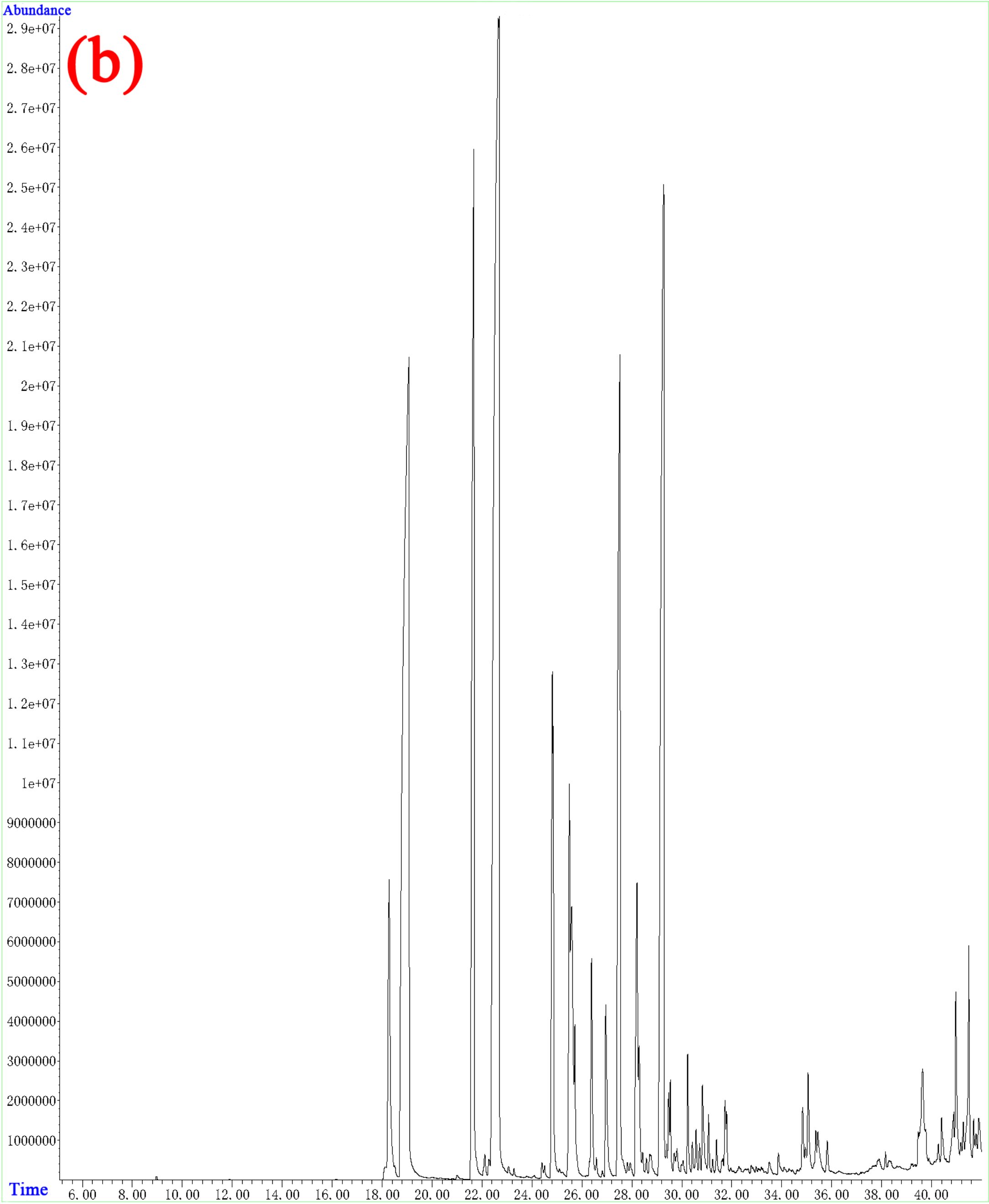

(b)
Abundance
2.9e+07
2.8e+07
2.7e+07
2.6e+07
2.5e+07
2.4e+07
2.3e+07
2.2e+07
2.1e+07
2e+07
1.9e+07
1.8e+07
1.7e+07
1.6e+07
1.5e+07
1.4e+07
1.3e+07
1.2e+07
1.1e+07
1e+07
9000000
8000000
7000000
6000000
5000000
4000000
3000000
2000000
1000000
Time
6.00
8.00
10.00
12.00
14.00
16.00
18.00
20.00
22.00
24.00
26.00
28.00
30.00
32.00
34.00
36.00
38.00
40.00

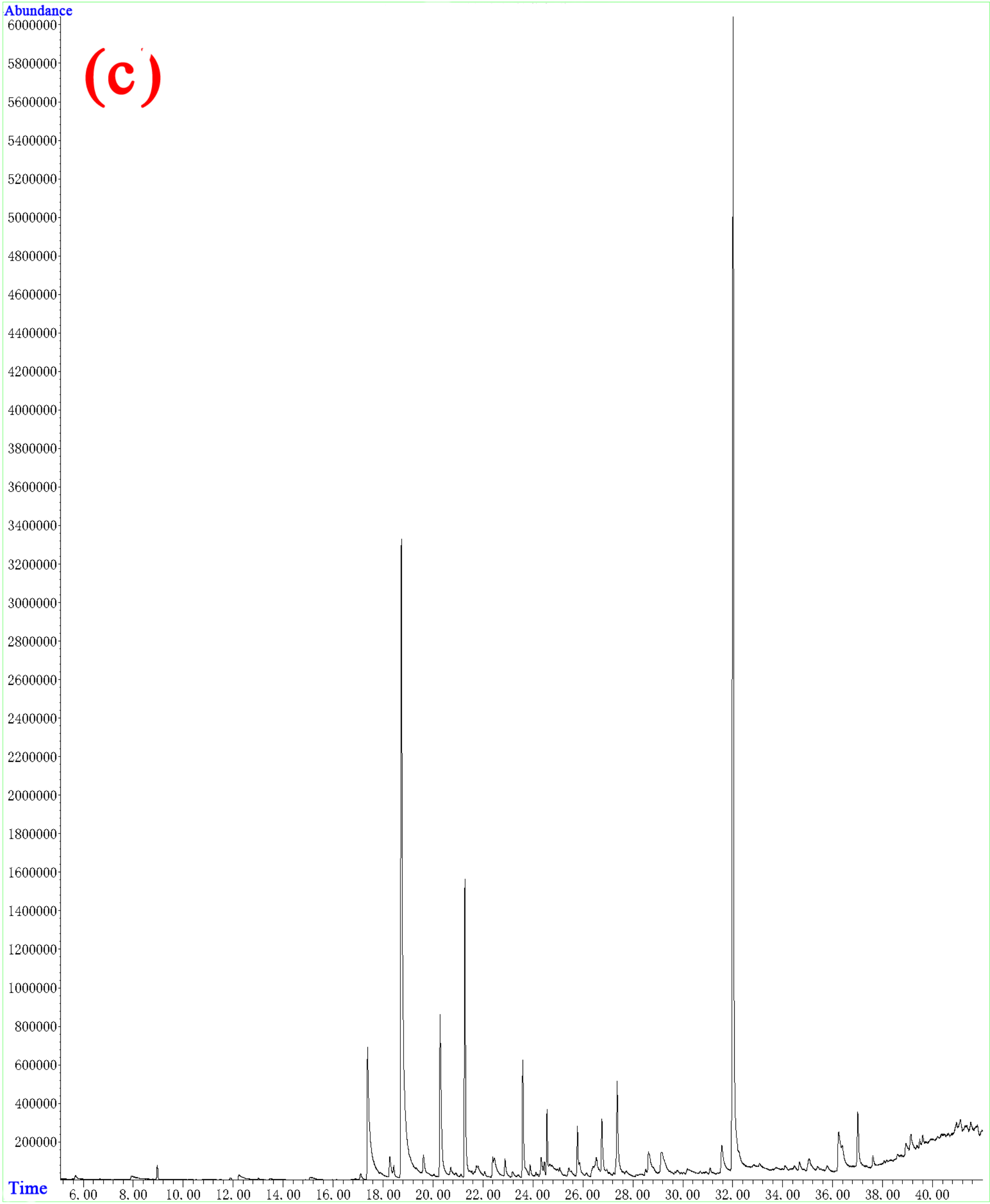

Abundance
(c)
6000000
5800000
5600000
5400000
5200000
5000000
4800000
4600000
4400000
4200000
4000000
3800000
3600000
3400000
3200000
3000000
2800000
2600000
2400000
2200000
2000000
1800000
1600000
1400000
1200000
1000000
800000
600000
400000
200000
Time
6.00
8.00
10.00
12.00
14.00
16.00
18.00
20.00
22.00
24.00
26.00
28.00
30.00
32.00
34.00
36.00
38.00
40.00

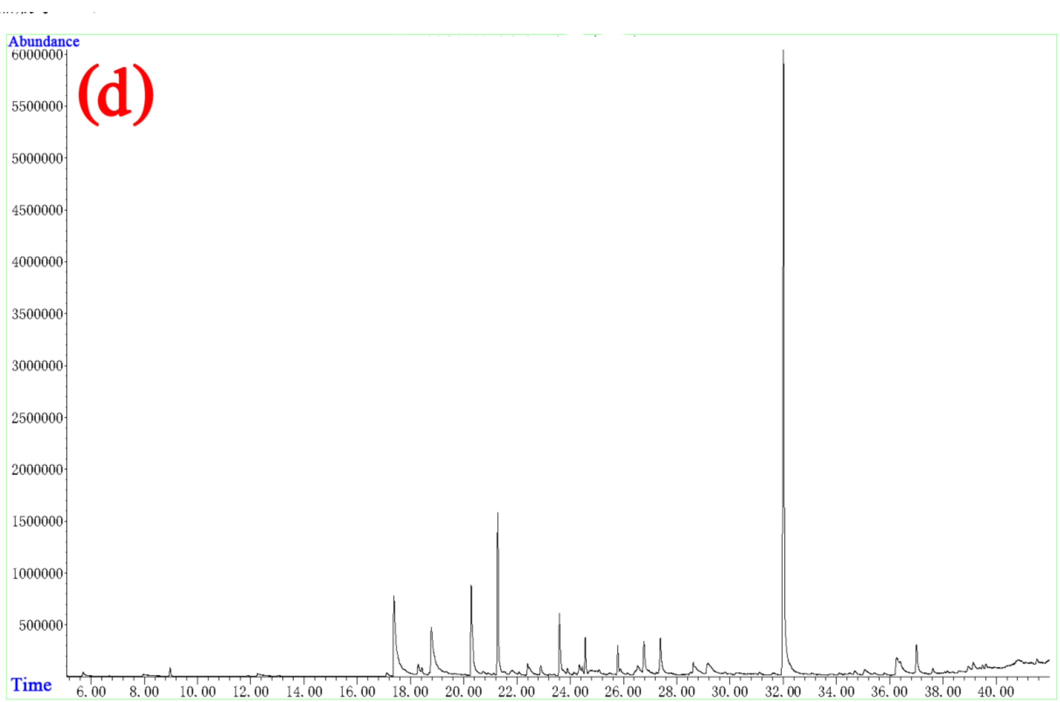

Fig. S1 GC–MS diagram of effluent from different processes: (a) raw water; (b) coagulation; (c) coagulation + O_3 catalytic oxidation; (d) coagulation + O_3 catalytic oxidation with addition of H_2O_2
